# Sr_2_MnO_2_Na_1.6_Se_2_: A Metamagnetic Layered Oxychalcogenide Synthesized by Reductive Na Intercalation to Break [Se_2_]^2–^ Perselenide Dimer Units

**DOI:** 10.1021/acs.chemmater.4c00801

**Published:** 2024-05-30

**Authors:** Souvik Giri, Sunita Dey, Emmanuelle Suard, Simon J. Clarke

**Affiliations:** † Department of Chemistry, University of Oxford, Oxford OX1 3QR, U.K.; ‡ Department of Chemistry, University of Aberdeen, Meston Walk, Aberdeen AB24 3UE, U.K.; § Institut Laue-Langevin (ILL), BP 156, 71 Avenue des Martyrs, Grenoble 38042, France

## Abstract

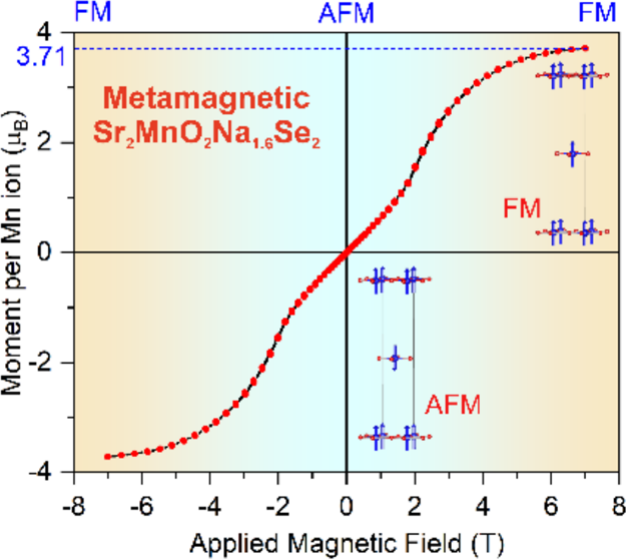

Recent advances in anion-redox topochemistry have enabled the synthesis of metastable mixed-anion solids. Synthesis of the new transition metal oxychalcogenide Sr_2_MnO_2_Na_1.6_Se_2_ by topochemical Na intercalation into Sr_2_MnO_2_Se_2_ is reported here. Na intercalation is enabled by the redox activity of [Se_2_]^2–^ perselenide dimers, where the Se–Se bonds are cleaved and a [Na_2–*x*_Se_2_]^(2+*x*)–^ antifluorite layer is formed. Freshly prepared samples have 16(1) % Na-site vacancies corresponding to a formal oxidation state of Mn of +2.32, a mixed-valence between Mn^2+^ (d^5^) and Mn^3+^ (d^4^). Samples are highly prone to deintercalation of Na, and over two years, even in an argon glovebox environment, the Na content decreased by 4(1) %, leading to slight oxidation of Mn and a significantly increased long-range ordered moment on the Mn site as measured using neutron powder diffraction. The magnetic structure derived from neutron powder diffraction at 5 K reveals that the compound orders magnetically with ferromagnetic MnO_2_ sheets coupled antiferromagnetically. The aged sample shows a metamagnetic transition from bulk antiferromagnetic to ferromagnetic behavior in an applied magnetic field of 2 T, in contrast to the Cu analogue, Sr_2_MnO_2_Cu_1.55_Se_2_, where there is only a hint that such a transition may occur at fields exceeding 7 T. This is presumably due to the higher ionic character of [Na_2–*x*_Se_2_]^(2+*x*)–^ layers compared to [Cu_2–*x*_Se_2_]^(2+*x*)–^ layers, reducing the strength of the antiferromagnetic interactions between MnO_2_ sheets. Electrochemical Na intercalation into Sr_2_MnO_2_Se_2_ leads to the formation of multiphase sodiated products. The work shows the potential of anion redox to yield novel compounds with intriguing physical properties.

## Introduction

Over the past decade, mixed-anion compounds with more than one anion (such as oxychalcogenides, oxyhalides, oxynitrides, etc.) have emerged as potential functional materials for a variety of applications from visible light photocatalysts to solid electrolytes to thermoelectrics.^1−5^ They offer several flexibilities over monoanionic compounds (such as oxides): unique heteroleptic coordination geometries, layered structures, and varying polarizabilities, to name a few.^6^ Heteroleptic coordination enables the tuning of the crystal field splitting of transition metal ions, and intergrowth structures induce two-dimensional (2D) character, leading to novel properties, such as high-*T*_c_ superconductivity.^7,8^

There is a large number of known oxychalcogenide compounds with the general formula Ae_2_MO_2_X_2–*x*_Ch_2_ (Ae = Sr, Ba; M = mid-to-late three-dimensional (3D) transition metal, except for Fe; X = Cu, Ag; Ch = S, Se, Te; 0 < *x* < 0.5) with M in a highly distorted octahedral coordination environment with a square of equatorial oxide ions and chalcogenides in the axial positions.^9^ Many of these show unique structural and physical properties. For example, Sr_2_MnO_2_Cu_2*m*–*x*_S_*m*+1_ (*m* = 1, 2, 3; *x* ∼ 0.5) is a homologous series of metamagnetic compounds,^10^ Ba_2_CoO_2_Cu_2_S_2_ has a large unquenched orbital moment on Co,^11,12^ and Ba_2_ZnO_2_Ag_2_Ch_2_ (Ch = Se, Te) have discrete [ZnO_2_]^2–^ linear units.^13,14^ This class of compounds with layered structures and low thermal conductivity are also investigated for potential thermoelectric applications.^15,16^ The structures of this class of compounds can be understood as an alternating stacking of [Ae_2_MO_2_]^(2+*x*)+^ perovskite-type layers and [X_2–*x*_Ch_2_]^(2+*x*)–^ antifluorite-type layers along the *c* axis. While the former of the two layers controls the magnetic properties, the latter is a playground for soft chemical manipulation. For instance, ∼10% of the Cu can be deintercalated from Sr_2_MnO_2_Cu_1.5_S_2_ using solvated I_2_ at ambient temperatures, leading to a change of the Cu-vacancy ordering scheme, oxidation of Mn, and a significant modification of the magnetic structure.^17^ A similar composition control of magnetic ordering has been achieved by Blandy et al., where deintercalation of ∼15% of the Cu from Sr_2_MnO_2_Cu_1.8_Te_2_ results in the transformation from a state with short-range 2D magnetic ordering to the one with 3D antiferromagnetic ordering.^18^ In contrast to the case of the oxysulfide and oxytelluride analogues, Cu cannot be deintercalated from the oxyselenide compound Sr_2_MnO_2_Cu_1.55_Se_2_^19^ using traditional reagents such as I_2_, likely due to slow kinetics. We recently reported that most of the Cu can be deintercalated from Sr_2_MnO_2_Cu_1.55_Se_2_ using a new synthetic path.^20^ First, the Cu in the chalcogenide layer was extruded from the structure as Cu metal using strongly reductive *n*-butyllithium (BuLi) intercalation, forming a high-energy reactive intermediate, Sr_2_MnO_2_Li_2_Se_2_, decorated by the extruded elemental Cu.^21^ Then, the S–S bond of disulfiram ((Et_2_NCS_2_)_2_) was used as an oxidant to dissolve the elemental Cu and chelate the resulting Cu^2+^ ions and also deintercalate Li from the selenide layers of the oxide selenide. This multistep route to net Cu deintercalation led to the activation of anion redox and collapse of the [Cu_1.5_Se_2_]^(2+*x*)–^ layer into a 2D array of [Se_2_]^2–^ perselenide dimers producing Sr_2_MnO_2_Se_2_.

Sasaki et al. recently proposed compounds containing these [Ch_2_]^2–^ (Ch = S, Se) dimers as intercalation hosts, which differ from traditional intercalation hosts such as 2D or 1D van der Waals systems.^22^ They can operate as a “zipper”. When reducing guest *A* is intercalated, the zipper opens via the reduction of dichalcogenide ions and the breaking of the Ch–Ch bonds, and the inserted A^+^ cations balance the charge. Reversible deintercalation leads to zipper closing by forming anion dimers. Some examples of this type of intercalation chemistry have been reported in recent literature. Sasaki et al. showed that Cu can be intercalated to reduce [S_2_]^2–^ persulfide dimers in La_2_O_2_S_2_ and Ba_2_F_2_S_2_ to form La_2_O_2_Cu_2_S_2_ and Ba_2_F_2_Cu_2_S_2_ products with [Cu_2_S_2_] layers, respectively.^23^ Similarly, Cu and Li can be intercalated to reduce the [Se_2_]^2–^ dimers in LaSe_2_ and Bi_2_O_2_Se_2_, producing LaCuSe_2_ and Bi_2_O_2_Li_2_Se_2_, respectively.^23,24^ Here, we extended the scope of “zipper” chemistry to the aforementioned complex compound Sr_2_MnO_2_Se_2_, where 2D perovskite-type slabs are interconnected by anionic molecular dimers. In contrast to the other examples where the dimers were present with redox-inactive layers, Mn acts as a potential redox center in this compound. We observed that Na intercalation in Sr_2_MnO_2_Se_2_ leads to the conversion of layers of [Se_2_]^2–^ dimers into [Na_2–*x*_Se_2_] antifluorite layers, forming Sr_2_MnO_2_Na_2–*x*_Se_2_ with *x* reaching a minimum value of 0.3, which is highly air-sensitive with facile partial deintercalation to obtain Sr_2_MnO_2_Na_1.6_Se_2_ (*x* = 0.4). Similar [Na_2–*x*_Ch_2_] antifluorite layers with Na in tetrahedral coordination by chalcogen atoms (Ch) in a layered intergrowth structure are uncommon. One known example is metastable β-NaY_2_Ti_2_O_5_S_2_ with [NaS_2_]^3–^ layers.^25^ Sr_2_MnO_2_Na_1.6_Se_2_ is the first reported example in the Ae_2_MO_2_X_2−δ_Ch_2_ family of compounds with Na in the chalcogenide layer and is a mixed-valent manganese compound with unusual magnetism that is metastable and requires a multistep route for synthesis.

## Experimental Methods

### Synthesis

The powder sample of Sr_2_MnO_2_Na_2–*x*_Se_2_ was synthesized in 0.1–1.5 g batches by reductive sodium intercalation into the collapsed selenide phase Sr_2_MnO_2_Se_2_. The parent material Sr_2_MnO_2_Se_2_ was prepared by the multistep process summarized above and as described in detail previously.^20^ Sodium intercalation was performed by stirring 0.2–1.5 g of suspended Sr_2_MnO_2_Se_2_ powder with a 3-fold molar excess of sodium naphthalenide (Na^+^[C_10_H_8_]^−^) in tetrahydrofuran (THF) solution for 5 days at room temperature in an inert atmosphere. Equivalent amounts of freshly cut sodium (Sigma-Aldrich, 99%) and naphthalene (Thermo Fisher Scientific, 99.6%) were dissolved and stirred overnight in anhydrous THF to produce the ∼0.15 M green sodium naphthalenide solution used in the reaction. The solution was transferred to a new Schlenk flask containing the oxide selenide powder using a cannula and stirred for the completion of the reaction. After the reaction, the black powder product was filtered and washed twice with fresh, dry THF to remove excess sodium naphthalenide. Then, the product was dried under dynamic vacuum before being transferred to a dry glovebox. The product was found to be highly air-sensitive, as described below, and all manipulations of solids were carried out in an argon-filled glovebox with an O_2_ level below 5 ppm.

### X-ray and Neutron Powder Diffraction

The solid product was first investigated by using an in-house Bruker D8 Advance Eco diffractometer (Cu Kα radiation). The sample, judged pure from laboratory X-ray diffraction, was measured on the synchrotron powder X-ray diffraction (SPXRD) beamline I11^26^ at the Diamond Light Source, U.K. The sample was thoroughly ground with amorphous dry silica glass to reduce sample absorption and sealed in a 0.5 mm diameter borosilicate capillary under argon. The SPXRD data was collected with Si-calibrated X-rays with a wavelength of approximately 0.826 Å (the exact wavelength for each measurement is mentioned in the Rietveld plots) using a position-sensitive detector (Mythen PSD) with a resolution of Δ*d*/*d* ≈ 10^–3^–10^–4^.

To obtain the exact structural parameters of light atoms (Na and O) and determine the magnetic structure of Sr_2_MnO_2_Na_1.6_Se_2_, neutron powder diffraction (NPD) data were collected on the same sample on the D2B diffractometer^27^ in October 2021 at the Institut Laue-Langevin (ILL), which is optimized for high-resolution powder diffraction, with a wavelength λ of 1.594 Å chosen using a Germanium [115] crystal as a monochromator. The powder sample (∼1.2 g) was loaded into a 6 mm diameter vanadium can and sealed with an indium wire in the glovebox. The sample was measured first at room temperature in the 2θ range from 5 to 160° over 4 h and then cooled to 2 K using a cryostat, and data were collected with a similar scan sequence. In November 2023, to characterize the metamagnetic nature of Sr_2_MnO_2_Na_1.6_Se_2_, the same sample (recovered from the 2021 measurement and stored in the meantime in an argon-filled glovebox) was measured on D2B both with and without a magnetic field. The sample (∼0.8 g) was loaded into a 6 mm diameter vanadium can in the glovebox, and the empty space in the can was packed with a Cd foil roll and Cd foil discs to prevent physical sample movement in a strong magnetic field (cadmium absorbs neutrons very strongly and makes a negligible contribution to the scattering). After the data collection at room temperature, the sample was cooled to 5 K in a cryomagnet with no magnetic field applied. The diffraction pattern was recorded over the 2θ range from 5 to 160° over 5 h. NPD under a 5.5 T vertical applied magnetic field was then collected by using a similar scan sequence. Two further scans were then collected after turning off the magnetic field while still at 5 K and after warming to 100 K, again without a field.

Quantitative structural parameters were obtained from Rietveld refinement against SPXRD and NPD data. The Rietveld refinements were performed using TOPAS Academic software.^28^ Pseudo-Voigt peak shapes appropriate to the instruments were used, and a Chebyshev polynomial was used to fit the backgrounds. All Rietveld refinements were performed using isotropic thermal displacement parameters (*B*_iso_). A high correlation between *B*_iso_(Na) and Na occupancy was observed for the NPD data. To reduce parameter correlations, *B*_iso_(Na) was constrained to twice the *B*_iso_(Se), which is in line with high Na mobility in the layer. The poor signal-to-noise ratio in the low-temperature NPD data compelled us to use a single *B*_iso_ for all of the atoms except for Na, as *B*_iso_(Na) was set to twice that value.

### Magnetometry

The magnetic response of the sample was recorded by using a Quantum Design MPMS3 SQUID magnetometer. Magnetization (*M*) against variable temperature (2–300 K) measurements was carried out on a 15 mg sample loaded into a gelatin capsule in an inert atmosphere. For this measurement, the sample was first cooled in zero field; a field of 100 Oe was then applied, and the magnetic moment of the sample was measured on warming (zero-field-cooled (ZFC) measurement). Then, the sample was cooled in the 100 Oe field and measured again on warming (field-cooled (FC) measurement). The susceptibility in the paramagnetic regime (150 K < *T* < 285 K) was fitted to the Curie–Weiss law (, where *C* is the Curie constant and θ is the Weiss temperature). Additionally, magnetization (*M*) versus magnetic field (*H*) isotherms were recorded at 2 K immediately after the ZFC/FC cycle of measurements after cooling the sample in the magnetic field of 100 Oe. Similar magnetic isotherm measurements were made immediately prior to the in-field NPD measurement.

### X-ray Absorption Spectroscopy (XAS)

Mn *K*-edge X-ray absorption spectroscopy was collected on the B18 beamline (Diamond Light Source, U.K.) in the energy range from 6340 to 7389 eV in transmission mode. Mn foil was used as a reference to calibrate and align the spectra. The samples were prepared as 13 mm diameter pellets by mixing 10–20 mg of each sample with ∼60 mg of microcrystalline cellulose. The pellets were loaded in the sample holder, which was sealed inside an aluminized polythene bag under an inert atmosphere to protect the samples. The data calibration, normalization, and analysis were performed using the Athena software package.^29^

### Electrochemistry

Electrochemical sodium intercalation and subsequent deintercalation were carried out on the oxidized selenide phase Sr_2_MnO_2_Se_2_^20^ in a coin cell assembly (CR2032, Cambridge Energy Solutions). All manipulations were performed in a glovebox under an argon atmosphere. Sr_2_MnO_2_Se_2_ was mixed with a poly(vinylidene fluoride) (PVDF) binder and Super-P conductive carbon (Timcal) in an 8:1:1 weight ratio to prepare the cathode composite. The coin cell was assembled with a cathode composite, a borosilicate glass fiber separator (Whatman, 15 mm diameter) soaked in 75 μL of the electrolyte (freshly prepared 1 M NaPF_6_ in propylene carbonate), and a Na counter electrode (Na metal was removed from the mineral oil, cleaned thoroughly with hexane, and cut into a disk with a 13 mm diameter and around 1 mm thickness). Galvanostatic (dis)charge was carried out at room temperature with a Lanhe battery cycler (Wuhan Land Electronics Co. Ltd.) at *C*/10 rate, where *C* is defined as the theoretical capacity of the active material (Sr_2_MnO_2_Se_2_) and *C*/10 means fully charging or discharging over 10 h. After discharging the sample to 0.5 V, the coin cell was disassembled in the glovebox, and the cathode material was washed with dimethyl carbonate (Sigma-Aldrich, 99% anhydrous) to remove the Na salt and dried in the glovebox antechamber under dynamic vacuum for 30 min. The powder sample was measured using SPXRD after being sealed in a borosilicate capillary.

## Results and Discussion

### Synthesis and Crystal Structure

**Figure 1:**
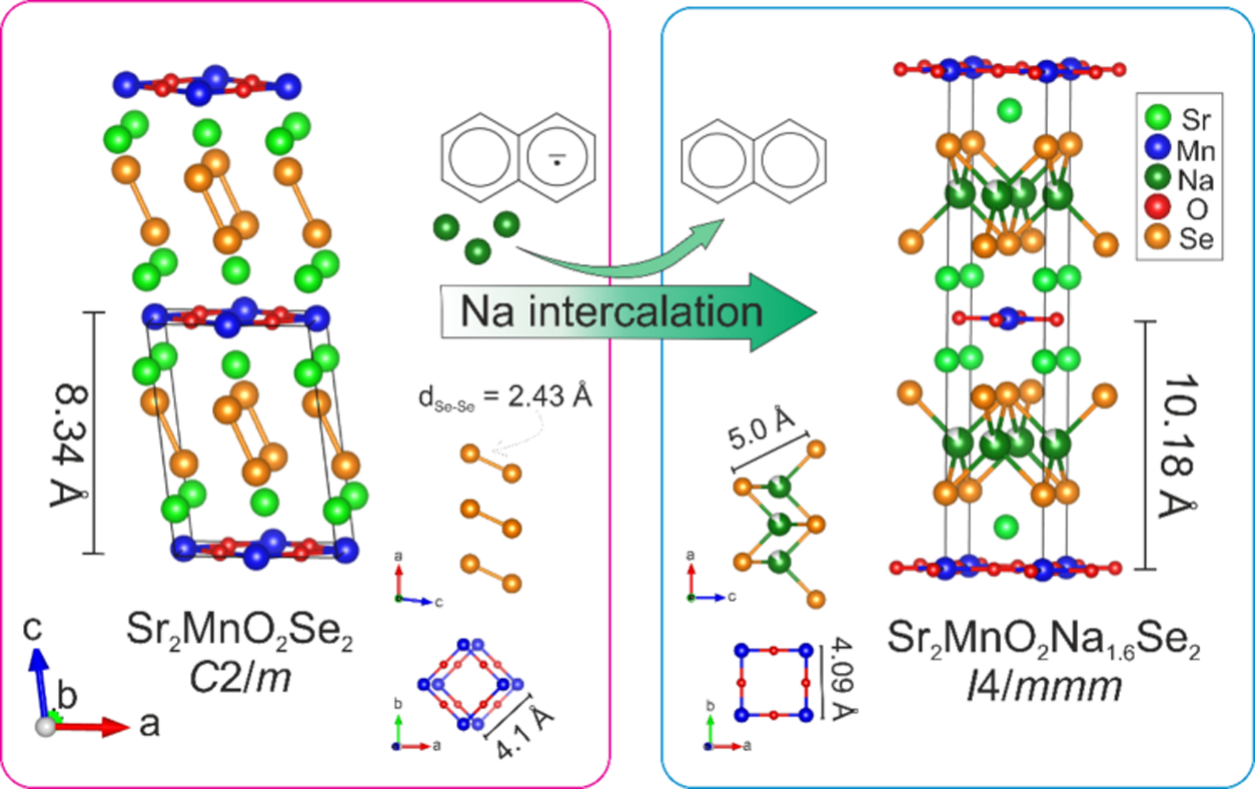
Schematic of topochemical Na intercalation into the Sr_2_MnO_2_Se_2_ structure with [Se_2_]^2–^ dimers, using Na^+^[C_10_H_8_]^−^ as a reductant. Na intercalation leads to the cleavage of the Se–Se bond, forming [Na_2–*x*_Se_2_] layers.

The novel compound Sr_2_MnO_2_Na_1.6_Se_2_ was synthesized using the anionic redox chemistry of the parent compound Sr_2_MnO_2_Se_2_.^20^ The structure of Sr_2_MnO_2_Se_2_ consists of [Se_2_]^2–^ dimers (bond length = 2.43 Å) in a two-dimensional (2D) array sandwiched between MnO_2_ square planar slabs (Figure 1). The MnO_2_ planes are offset from each other along the vector 0.3*a* + 0.3*b* (*a* and *b* are the basal lattice parameters), giving the structure monoclinic symmetry. The Mn cations are also weakly coordinated in each axial direction by one end of a [Se_2_]^2–^ anion with a Mn–Se distance of 3.079 (1) Å.

Sodium naphthalenide (Na^+^[C_10_H_8_]^−^) is a common reducing agent in organic and organometallic chemistry with a reduction potential of −2.5 V versus the normal hydrogen electrode (NHE), where the radical naphthalenide species acts as the redox center.^30^ Upon reaction with sodium naphthalenide, electrons are introduced into the empty antibonding σ* orbital of [Se_2_]^2–^, leading to the cleavage of the dimers and formation of [Na_2_Se_2_]^2–^ edge-sharing tetrahedra, giving the Sr_2_MnO_2_Cu_1.5_S_2_ structure type (Figure 1).^10^ As shown below, the Mn–O bond length shows little change from the parent compound to the product. This implies that the Mn oxidation state change is small, and the bulk of the redox process involves the anionic redox of Se dimers. No impurity phase was found in the diffraction pattern, which highlights the effectiveness of sodium naphthalenide as a selective and powerful reducing agent. Sr_2_MnO_2_Na_1.6_Se_2_ with more oxophilic Na in the selenide layer and more chalcophilic Mn in the oxide layer cannot be produced from traditional high-temperature solid-state synthesis from binary compounds, hinting at its metastable nature, as discussed further in Figure S1.

**Figure 2:**
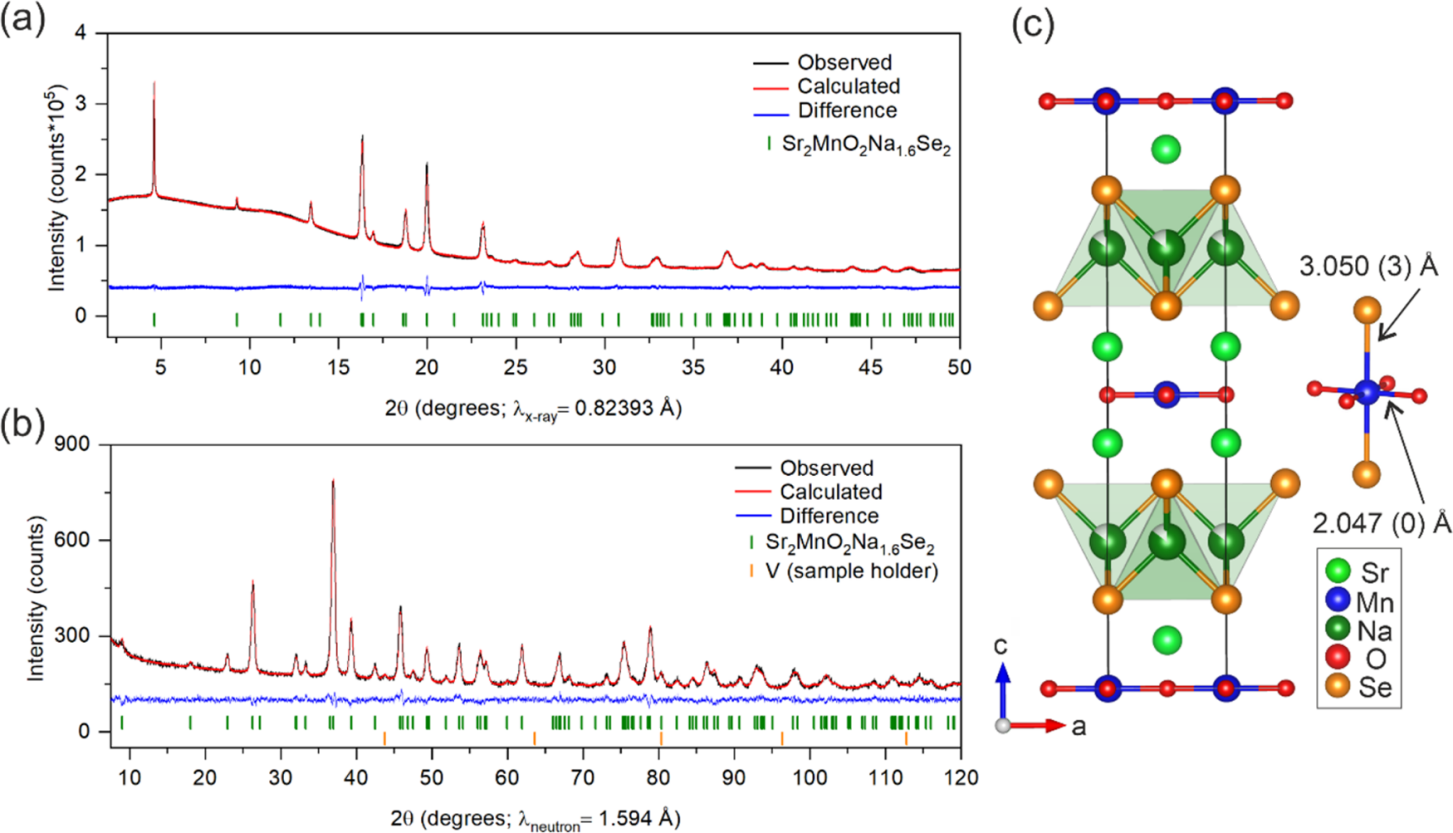
Room-temperature Rietveld refinement of Sr_2_MnO_2_Na_1.6_Se_2_ against (a) SPXRD and against (b) NPD (2023). (c) Refined crystal structure obtained from the Rietveld refinement against 2023 NPD data. The highly distorted Mn coordination environment is shown on the right. Isotropic displacement parameters for all atoms are shown at 99% probability, as extracted from the Rietveld refinement against NPD data.

The structure was confirmed by Rietveld refinement against both SPXRD and NPD data at room temperature. As mentioned in the Experimental Methods section, the same sample was measured twice using NPD (in 2021 and 2023). The 2023 Rietveld refinements on the aged sample are depicted in Figure 2, and the refined structural parameters are listed in Table 1 (see Figure S2 for Rietveld refinement against 2021 NPD at room temperature). All data show the compound Sr_2_MnO_2_Na_1.6_Se_2_ crystallizes in the space group *I*4/*mmm* (No. 139). It is isostructural with layered Sr_2_MnO_2_Cu_1.55_Se_2_ with Na^+^ ions formally replacing the Cu^+^ ions in the chalcogenide layer (to be noted: the parent compound Sr_2_MnO_2_Se_2_ was prepared from Sr_2_MnO_2_Cu_1.55_Se_2_ by a multistep Cu deintercalation process via a lithiated intermediate^20^). Due to the intercalation of Na ions, the interlayer spacing between two MnO_2_ planes increased by 22.0% (*d*_interlayer, Sr_2_MnO_2_Se_2__ = 8.340(2) Å; *d*_interlayer, Sr_2_MnO_2_Na_1.6_Se_2__ = 10.1747(5) Å). The size difference of Na^+^ and Cu^+^ (*r*_Na+_ = 0.99 Å; *r*_Cu+_ = 0.6 Å^31^) is manifested in the 13.8% larger *c* lattice parameter of Sr_2_MnO_2_Na_1.6_Se_2_ compared to the Cu analogue (*d*_interlayer, Sr_2_MnO_2_Cu_1.55_Se_2__ = 8.94150(5) Å). As a result of expansion along the *c* direction and limited expansion in the *ab* plane, the NaSe_4_ tetrahedra are heavily distorted compared to the CuSe_4_ tetrahedra in Sr_2_MnO_2_Cu_1.55_Se_2_ (see Table 2). Rietveld refinement from the 2021 NPD experiment revealed that the Na site in the chalcogenide layer is 16(1) % vacant, which is common in these Mn oxychalcogenide compounds: in the Sr_2_MnO_2_Cu_2−δ_Ch_2_ (Ch = S, Se, Te) composition space, the sulfide and selenide have about 25% of the Cu sites vacant, whereas the telluride has only 9.1% of the Cu sites vacant.^18,32^ The Cu vacancy in the telluride can be increased to 21% by topochemical Cu deintercalation with I_2_ in an acetonitrile solution. This cation vacancy concentration directly affects the formal Mn oxidation state. 25% site vacancy in the Cu layer indicates that the concentrations of Mn^2+^ and Mn^3+^ are equal. In the fresh sample of Sr_2_MnO_2_Na_2–*x*_Se_2_ measured using NPD in 2021, 16(1) % Na vacancy suggests a higher concentration of Mn^2+^ with a refined composition Sr_2_MnO_2_Na_1.7_Se_2_ (refined composition of Sr_2_MnO_2_Na_1.68(2)_Se_2_). The 2023 NPD data measured on the same sample revealed that despite the storage of the sample in a dry argon glovebox environment, the Na occupancy evolved slightly over time and the site was 20(1) % vacant, indicating a 4(1) % loss of Na by oxidative deintercalation and a composition Sr_2_MnO_2_Na_1.6_Se_2_ (refined composition of Sr_2_MnO_2_Na_1.58(2)_Se_2_). This real change in the sample is reflected in a decrease in unit cell volume between the two measurements, which are two years apart (Table 1). Presumably, a small amount of Na is lost by reaction over many months with a small amount of O_2_/H_2_O present in the glovebox atmosphere or during handling immediately prior to measurements. No extra reflections were seen in neutron or X-ray powder diffraction on the aged sample corresponding to a Na-containing impurity phase (for example, NaOH), suggesting that the decomposition byproduct is amorphous in nature. The decrease in Na occupancy also corresponds to an increase in the Mn oxidation state, bringing it closer to +2.5 (aged sample formal Mn oxidation state = +2.4) than in the freshly made sample (formal Mn oxidation state = +2.3). As a result of this partial Mn oxidation, the *a* lattice parameter, which is double the Mn–O bond length, decreases and the *c*/*a* ratio increases. This is consistent with the oxidation of Mn or Co by Cu deintercalation observed in related compounds.^11,18^ The estimated Mn bond valence change based on the 0.05 Å shortening of the Mn–O distances is about 0.1, consistent with the change in the oxidation state based on the refined Na occupancy. The high vacancy concentration in the metal site in the chalcogenide layer also opens the possibility of the long-range ordering of vacancies at low temperatures as found for Sr_2_MnO_2_Cu_1.5_S_2._^32^ However, Sr_2_MnO_2_Na_1.6_Se_2_ does not show any new Bragg reflections arising from this at 100 K in SPXRD measurements (see Figure S3 for Rietveld refinement) nor in the low-temperature PND measurements. This is similar to its Cu analogue, Sr_2_MnO_2_Cu_1.55_Se_2_, where there is only evidence for short-range Cu/vacancy ordering seen in electron diffraction but no superstructure peaks in SPXRD measurements at low temperatures.^32^ We cannot rule out a similar short-range ordering phenomenon in this case. The structural parameters of the aged sample Sr_2_MnO_2_Na_1.6_Se_2_ are compared with those of the Cu and Ag analogues in Table 2.

**Table 1:** Structural Parameters at Room Temperature for a Single Evolving Sample of Sr_2_MnO_2_Na_2–*x*_Se_2_^a^,^b^

radiation and wavelength	synchrotron X-ray, 0.82393 Å	neutron, 1.594 Å	neutron, 1.594 Å
date of measurement	May, 2023	November, 2023	October, 2021
space group	*I*4/*mmm* (No: 139)		
*a* (Å)	4.10529(4)	4.0949(1)	4.1464(1)
*c* (Å)	20.3419(4)	20.349(1)	20.319(1)
*V* (Å^3^)	342.83(1)	341.23(3)	349.36(4)
*c/a*	4.9550(1)	4.9694(3)	4.9004(3)
*z*(Sr)^b^	0.08308(5)	0.08239(1)	0.0812(1)
*z*(Se)^b^	0.15088(5)	0.1498(1)	0.1519(1)
Na occupancy	0.829(4)	0.79(1)	0.84(1)
refined composition	Sr_2_MnO_2_Na_1.66(1)_Se_2_	Sr_2_MnO_2_Na_1.58(2)_Se_2_	Sr_2_MnO_2_Na_1.68(2)_Se_2_
*B*_iso_(Sr) (Å^2^)	0.78(1)	0.55(5)	0.88(8)
*B*_iso_(Mn) (Å^2^)	0.78(1)	0.4(1)	1.1(1)
*B*_iso_(O) (Å^2^)	0.78(1)	0.78(6)	0.97(8)
*B*_iso_(Na) = *B*_iso_(Se) × 2 (Å^2^)	1.56(2)	2.19(8)	2.3(1)
*B*_iso_(Se) (Å^2^)	0.78(1)	1.10(4)	1.15(5)
*R*_wp_ (%)	1.14	2.78	3.5
χ^2^	3.33	1.18	1.16

a A single sample was used for all these experiments, but they were carried out at different times, as indicated. The changes in lattice parameters, in particular the *c*/*a* ratio, are real and not due to differences in the experimental setup. They evidently arise from small changes in the Na content of the sample from Sr_2_MnO_2_Na_1.7_Se_2_ to Sr_2_MnO_2_Na_1.6_Se_2_ between the measurements, as reflected in the results from the neutron refinements.

b Sr, 4*e* (0,0,*z*); Mn, 2*a* (0,0,0); O, 4*c* (1/2,0,0); Na, 4*d* (1/2,0,1/4); Se, 4*e* (0,0,*z*).

**Table 2:** Comparison of the Selected Structural Parameters of Sr_2_MnO_2_X_2–*x*_Se_2_ (X = Cu, Ag, Na)

	Sr_2_MnO_2_Cu_1.55_Se_2_	Sr_2_MnO_2_Ag_1.54_Se_2_	Sr_2_MnO_2_Na_1.6_Se_2_
reference	(32)	(33)	this work
radiation	neutron	synchrotron X-ray	neutron, 2023
*a* (Å)	4.06655(3)	4.08798(1)	4.0949(1)
*c* (Å)	17.8830(1)	19.13391(9)	20.349(1)
*c*/*a* ratio	4.39759(4)	4.06853(3)	4.9694(3)
*V* (Å^3^)	295.729(5)	319.759(3)	341.23(3)
occupancy of *A*^+^ site	0.773(2)	0.770(3)	0.79(1)
interlayer separation (Å)	8.9415(1)	9.5669(1)	10.1745(5)
Mn–O distance (Å) [4]^a^	2.03328(1)	2.04399(1)	2.04732(8)
Mn–Se distance (Å) [2]^a^	3.0002(3)	2.9652(5)	3.050(3)
Mn–Se/Mn–O ratio	1.4779(5)	1.4507(5)	1.490(1)
*A*–Se distance (Å) [4]^a^	2.5094(2)	2.7357(3)	2.888(1)
Se–*A*–Se angle (deg) [2]^a^	108.25(5)	96.69(2)	90.29(7)
Se–*A*–Se angle (deg) [4]^a^	110.09(5)	116.215(9)	119.83(4)

a Numbers in square brackets give the number of bonds/angles of each type.

### Magnetic Properties

**Figure 3:**
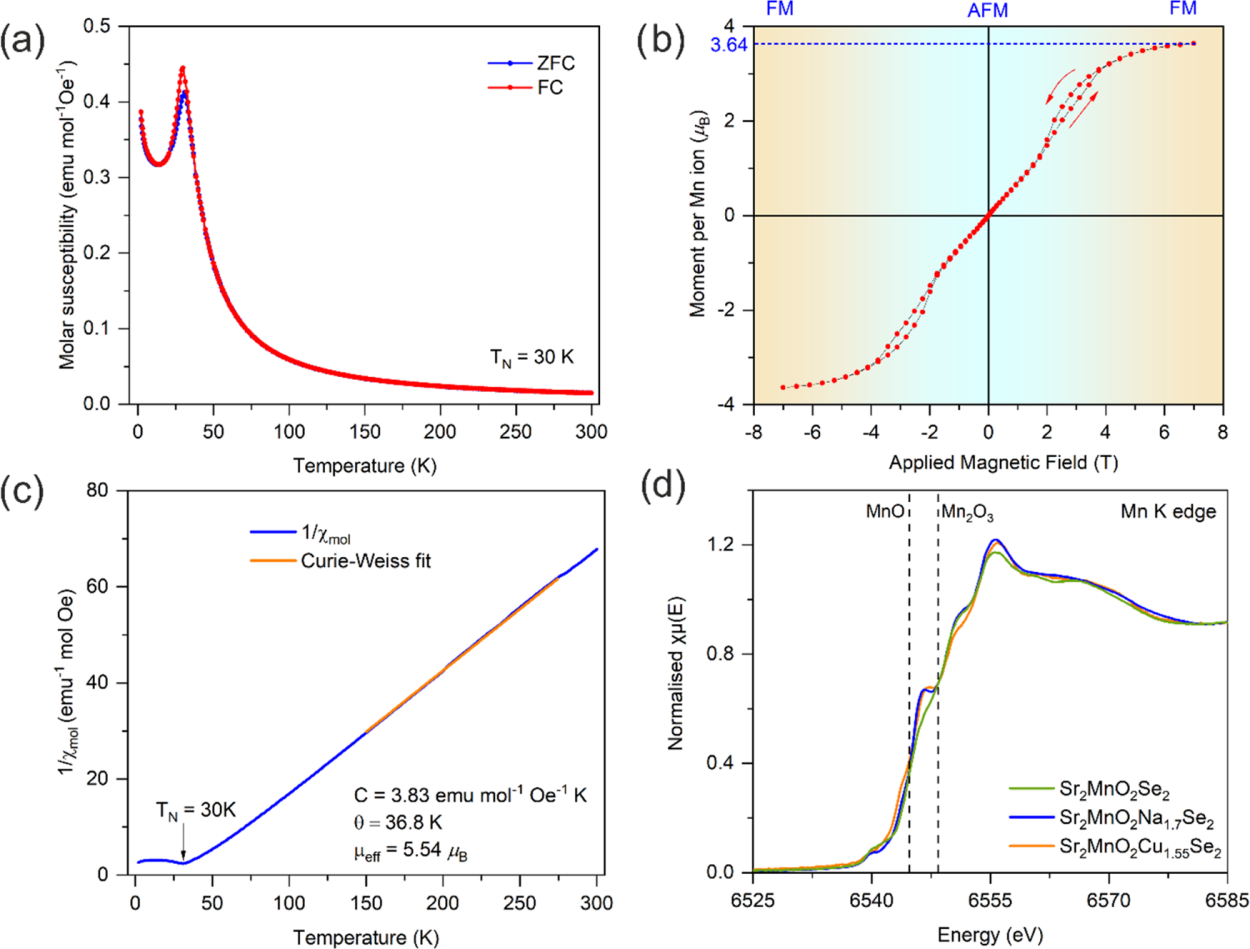
(a) Temperature dependence of the molar magnetic susceptibility of the fresh sample, Sr_2_MnO_2_Na_1.7_Se_2_, under ZFC/FC conditions measured in an applied field of 100 Oe (see the Experimental Section). (b) Magnetization versus magnetic field isotherms at 2 K showing a metamagnetic character with a saturated ferromagnetic moment of 3.64 μ_B_ per Mn ion at 7 T. The different colors represent different magnetic ground states. (c) Curie–Weiss fit to the linear region of the inverse susceptibility versus temperature curve. The extracted effective moment for Mn is 5.54 (3) μ_B_, which is between those of Mn^2+^ and Mn^3+^. (d) Mn *K*-edge XAS spectrum of Sr_2_MnO_2_Na_1.7_Se_2_ compared with its parent Sr_2_MnO_2_Se_2_ and its Cu analogue, Sr_2_MnO_2_Cu_1.55_Se_2_, showing that the Mn oxidation state and coordination are similar in all three compounds. This further confirms the intermediate Mn oxidation state in the compound, as the absorption edge is between those of MnO and Mn_2_O_3_.

**Figure 4:**
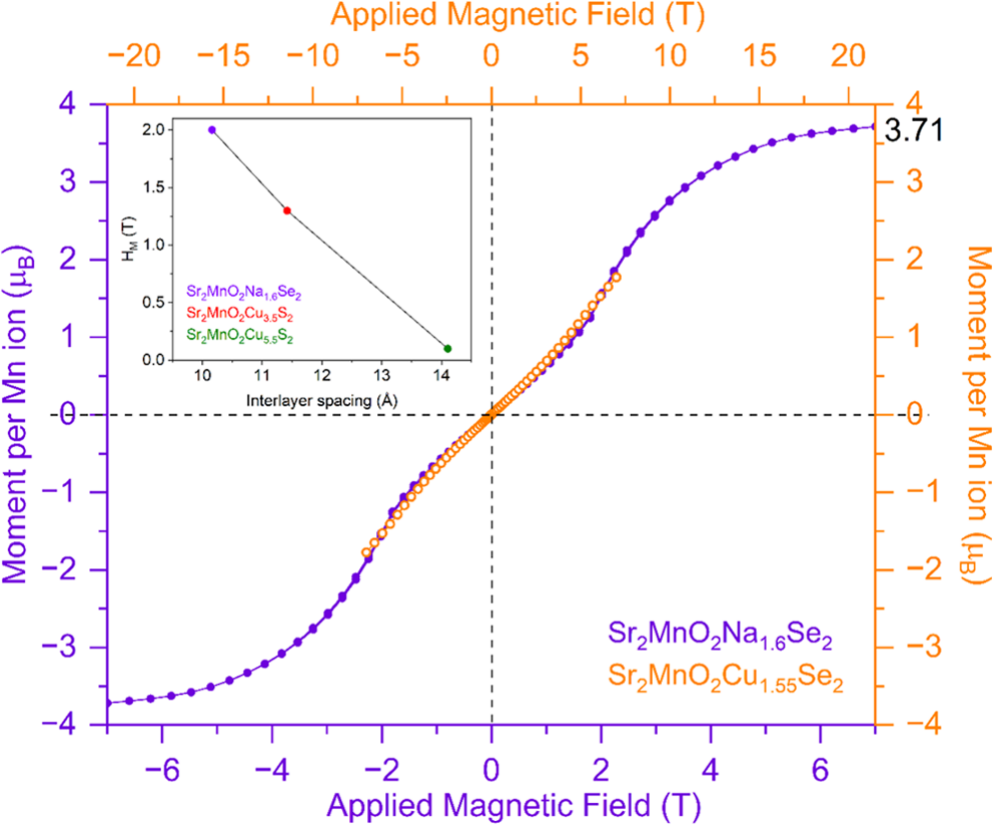
Comparison of magnetization (*M*) versus magnetic field (*H*) isotherms at 2 K for the aged sample, Sr_2_MnO_2_Na_1.6_Se_2_, and its copper analogue Sr_2_MnO_2_Cu_1.55_Se_2_. Although Sr_2_MnO_2_Na_1.6_Se_2_ exhibits an antiferromagnetic (AFM)-to-ferromagnetic (FM) transition at 2 T, the corresponding Cu compound Sr_2_MnO_2_Cu_1.55_Se_2_ does not enter the ferromagnetic regime up to 7 T, although at such fields, there is a slight upturn in the magnetization at high fields (see Figure S5). In the plot, the horizontal magnetic field axes have been scaled by overlaying the two plots. It suggests that Sr_2_MnO_2_Cu_1.55_Se_2_ may enter the ferromagnetic state at higher fields, and we estimate that it would approach saturation at 20 T. The inset shows inverse dependence of the field required for the metamagnetic transition (*H*_M_) with the spacing between two adjacent MnO_2_ layers.

The magnetic properties were measured in 2021 (Figure 3) and 2023 (Figure 4) and were found to be similar. Although we loaded the sample from the glovebox into the magnetometer in a sealed gelatin capsule, we cannot rule out that the slight oxidation to Sr_2_MnO_2_Na_1.6_Se_2_ occurred prior to the measurement. This would be consistent with the in-field NPD measurements discussed below. The magnetic ion Mn with a formal oxidation state of +2.4 in Sr_2_MnO_2_Na_1.6_Se_2_ has a highly distorted octahedral coordination (*d*_Mn–O equatorial_ = 2.04732 (8) Å and *d*_Mn–Se axial_ = 3.050 (3) Å; Figure 2c). The zero-field-cooled (ZFC) and field-cooled (FC) temperature-dependent magnetic susceptibility data of the fresh sample Sr_2_MnO_2_Na_1.7_Se_2_ are shown in Figure 3a. The magnetic response of the sample is similar to that of a typical antiferromagnetic (AFM) compound with a cusp in the magnetic susceptibility at a Néel temperature of 30 K. Very minimal divergence of ZFC and FC curves suggests the absence of spin domain formation. The low-temperature increase in susceptibility is the signature of a Curie tail presumed to be due to the presence of small amounts of paramagnetic impurities from the synthesis. The Curie–Weiss fit (Figure 3c) to the high-temperature data well above the transition yielded μ_eff_ = 5.54 (3) μ_B_ and a Weiss temperature of θ = 36.8 (2) K. The extracted magnetic moment supports a formal oxidation state of Mn^2.3+^, and the positive Weiss temperature indicates dominant ferromagnetic (FM) interactions. This is expected, as oxygen-mediated superexchange interactions between nearest-neighbor Mn^2+^ (d^5^) and Mn^3+^ (d^4^) via the d_*x*^2^–*y*^2^_ orbitals in this environment are predominantly ferromagnetic from the Goodenough–Kanamori rules.^34^ The XAS data (Figure 3d) further confirm the intermediate oxidation state, as the sample absorption edge position is intermediate between those of MnO and Mn_2_O_3_ and is similar to that of Sr_2_MnO_2_Cu_1.55_Se_2_. The dotted line in Figure 3d represents the maxima of the first peak of the d(χμ(*E*))/d*E* plot of MnO and Mn_2_O_3_ (see ref (10) for their spectra).

The magnetization (*M*) versus field (*H*) isotherm (Figure 3b) for the fresh sample, Sr_2_MnO_2_Na_1.7_Se_2_, at 2 K reveals a metamagnetic response of the sample at relatively low fields (see Figure S4 for *M* vs *H* at room temperature). This means that at stronger magnetic fields (*H* > 2 T), the antiferromagnetic interaction between the layers can be overcome, and spins can be flipped to achieve a fully ferromagnetic state. This metamagnetic behavior at low fields has also been observed in related compounds with thicker copper sulfide layers Sr_2_MnO_2_Cu_2*m*–*x*_S_*m*+1_ (*m* = 2,3; *x* ∼ 0.5)^10^ and recently in manganese oxychloride Ca_2_MnO_3_Cl^35^ with Mn^3+^. The AFM-to-FM transition can be achieved at magnetic fields of 1.3 T for the *m* = 2 compound Sr_2_MnO_2_Cu_3.5_S_3_ with *d*_interlayer spacing_ = 11.4180 (3) Å and 0.1 T for the *m* = 3 compound Sr_2_MnO_2_Cu_5.5_S_4_ with *d*_interlayer spacing_ = 14.107 (1) Å. The *m* = 1 compounds of the homologous series Sr_2_MnO_2_Cu_2–*x*_Ch_2_ (Ch = S, Se) do not enter the ferromagnetic regime at magnetic fields up to 7 T. This suggests that the critical field for the metamagnetic transition (*H*_M_) is inversely related to the interlayer separation between adjacent MnO_2_ sheets (Figure 4). Intriguingly, Sr_2_MnO_2_Na_2–*x*_Se_2_ (*x* = 0.3, 0.4), though being an *m* = 1 member of the series with *d*_interlayer spacing_ ≈ 10.17 Å, shows the AFM-to-FM transition at 2 T. Figure 4 shows the *M* versus *H* isotherm for the aged sample, Sr_2_MnO_2_Na_1.6_Se_2_, which is similar to the fresh sample, Sr_2_MnO_2_Na_1.7_Se_2_ (Figure 3b) in terms of the general shape of the isotherm and the saturation moment, but a small hysteresis in the metamagnetism was suppressed. Figure 4 also compares the 2 K magnetization isotherm of Sr_2_MnO_2_Na_1.6_Se_2_ with that of its Cu analogue, Sr_2_MnO_2_Cu_1.55_Se_2_, where it can be seen that the shape of the small upturn in magnetization at around 7 T for Sr_2_MnO_2_Cu_1.55_Se_2_ is similar to the behavior of Sr_2_MnO_2_Na_1.6_Se_2_ at around 2 T (see Figure S5 for the *M* vs *H* isotherm for Sr_2_MnO_2_Cu_1.55_Se_2_). The difference in magnetic response is plausibly due to the higher ionic character of Na^+^ compared to that of Cu^+^, which results in weaker AFM interactions between adjacent FM MnO_2_ sheets, leading to a lower critical field for the AFM-to-FM transition. This is also reflected in the Néel temperature for 3D long-range magnetic ordering of the compound, which is significantly lower than that of the Cu analogue. The comparison of the magnetic properties of Sr_2_MnO_2_X_2–*x*_Se_2_ (*X* = Cu, Ag, Na) is summarized in Table 3. The Weiss temperatures are similar, suggesting that the mean strength of the exchange interactions, which will be dominated by the in-plane interactions, is similar.

**Table 3:** Comparison of the Magnetic Properties of Sr_2_MnO_2_X_2–*x*_Se_2_ (X = Cu, Ag, Na)

	Sr_2_MnO_2_Cu_1.55_Se_2_	Sr_2_MnO_2_Ag_1.54_Se_2_	Sr_2_MnO_2_Na_1.7_Se_2_
reference	(32)	(33)	this work
Neel temperature, *T*_N_ (K)	53	63	30
Weiss temperature, θ (K)	43(1)	45(3)	36.8(2)
effective moment from the Curie–Weiss fit (μ_B_)	5.4(1)	5.45(1)	5.53(3)
magnetic structure	A-type AFM	A-type AFM	A-type AFM
critical field for the AFM-to-FM transition, *H*_M_ (T)	>7	>7	2

### Magnetic Structure

In the low-temperature NPD measurements (in 2021 and 2023), the appearance of extra Bragg reflections at high *d*-spacing indicated 3D antiferromagnetic ordering. The low-temperature neutron data collected in 2021 and 2023 are quite different (see Figure S6 for comparison). The magnetic peaks in the 2021 data are significantly broader and lower in intensity than in the 2023 data, confirming that the sample has evolved. As discussed previously, the refined Na occupancy from the 2021 data is 0.84(1) (formal Mn oxidation state = +2.32) compared to 0.79(1) (formal Mn oxidation state = +2.42) measured in 2023. This means the fresh Na-rich sample in 2021 possessed more Mn^2+^ than the aged Na-poor sample measured in 2023, which leads to increased disorder in the MnO_2_ plane between Mn^2+^ and Mn^3+^ as the formal oxidation state deviates further from +2.5 as a function of metal content in the chalcogenide layer. This departure from a Mn^3+^/Mn^2+^ ratio of 1:1 and the resultant introduction of some disorder into the magnetic structure might also be the reason for the broadening of the magnetic Bragg peaks. The magnetic scattering of the 2021 sample at 2 K was indexed on a √2*a* × √2*a* × *c* expansion of the nuclear cell. The initial space group was chosen as *P*1 to consider all possible modes. It was found that a single magnetic mode failed to account for all of the magnetic peaks. For example, activating the mM3+ mode, which is responsible for ferromagnetic coupling (moments perpendicular to the *ab* plane) between Mn ions within the MnO_2_ sheets and antiferromagnetic interaction between the adjacent MnO_2_ sheets along the stacking axis, could fit the magnetic peak intensity of (104) and (112) reflections at 2θ = 24° but was lacking intensity in the (100) and (004) reflections. These magnetic peaks could be modeled with the mX3+ mode, which results in antiferromagnetic Mn moments parallel to the *ab* plane, but this mode alone could not fit all of the magnetic reflections. Thus, a combination of mM3+ and mX3+ modes was needed to achieve a good visual and statistical fit. The magnetic structure can be described with the magnetic space group *P*4_2_′/*ncm*′ (138.523) in the Belov, Neronova, and Smirnova (BNS) scheme^29^ which accommodates these two modes. The refined magnetic structure consists of ferromagnetic MnO_2_ planes with Mn moments tilted away from the crystallographic *c* axis, with the planes coupled antiferromagnetically. The refined long-range ordered moment on the Mn ions is 2.04 μ_B_, which is significantly smaller than the refined magnetic moment from the 2023 NPD data (as described below). This reduced moment is presumably because of the higher compositional disorder between Mn^2+^ and Mn^3+^ in the MnO_2_ plane, as discussed above. The Rietveld refinement and the refined magnetic structure from low-temperature 2021 NPD data are shown in Figure S7 and Table S1.

**Figure 5:**
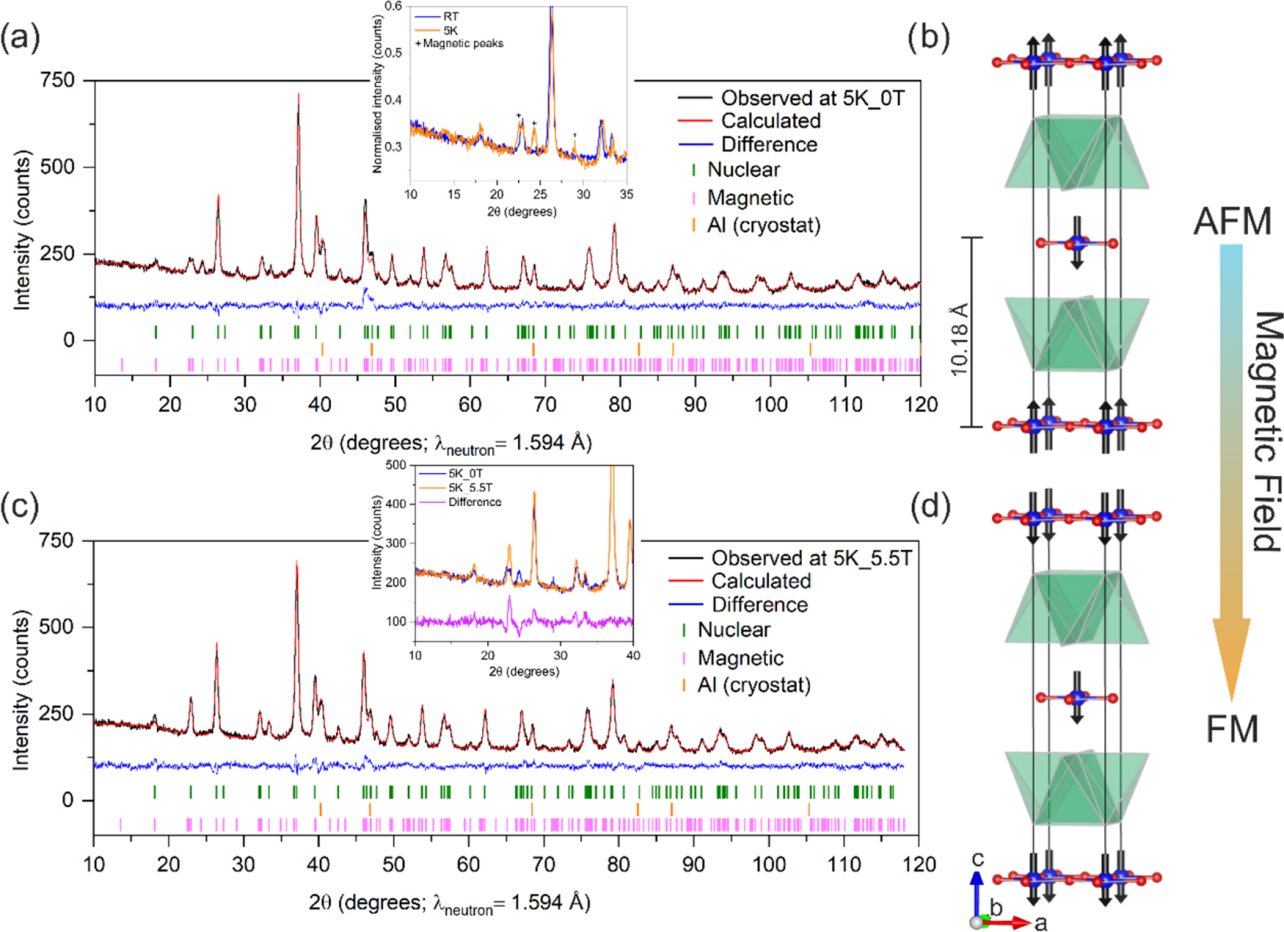
(a) Rietveld refinement against NPD data at 5 K (2023 measurement) without magnetic field *R*_wp_ = 3.47% and χ^2^ = 1.3. Inset: highlighting the appearance of magnetic Bragg reflections at 5 K compared with the room-temperature pattern. (b) Magnetic structure of Sr_2_MnO_2_Na_1.6_Se_2_ at zero magnetic field where Mn moments interact ferromagnetically within MnO_2_ planes and antiferromagnetically between two adjacent MnO_2_ planes. (c) Rietveld refinement against PND data at 5 K at a 5.5 T magnetic field. *R*_wp_ = 3.07%, and χ^2^ = 1.34. The inset highlights the disappearance of magnetic Bragg reflections and the emergence of magnetic intensity on top of the nuclear peaks. (d) The magnetic structure of Sr_2_MnO_2_Na_1.6_Se_2_ in a 5.5 T magnetic field, where the compound has undergone the metamagnetic transition and is in the ferromagnetic regime.

In the low-temperature NPD measurement collected in 2023, the magnetic peaks were narrower than those in the 2021 data but were still significantly broader than the nuclear ones, suggesting a slightly reduced coherence length for magnetic order compared with nuclear order. This is in line with disorder in magnetic interactions in the MnO_2_ plane. The new reflections can be indexed using the nuclear cell dimensions but without body centering. A magnetic phase with the space group *P*_*I*_4/*mnc* (128.410) in the BNS scheme^36^ accounted for the magnetic intensity and yielded a refined long-range ordered magnetic moment of 3.79 (9) μ_B_ per Mn ion at 5 K (see Table S2). In contrast to the 2021 data, all of the magnetic intensities can be fitted by activating only the mM3+ mode, which is present in this magnetic space group. Attempts to fit the magnetic peaks to alternative models where the interaction between Mn ions in the plane was antiferromagnetic failed. The Rietveld refinement against the NPD data at 5 K and the resultant magnetic unit cell are depicted in Figure 5a,b. This so-called A-type AFM structure is observed in related compounds with the formula Sr_2_MnO_2_X_2–*x*_Se_2_ (X = Cu and Ag; *x* ∼ 0.5) with a formal Mn oxidation state very close to +2.5 where the mixed-valence leads to in-plane ferromagnetic coupling, as discussed above. The refined long-range ordered moment per Mn ion of 3.79(9) μ_B_ from the 2023 data in Sr_2_MnO_2_Na_1.6_Se_2_ is comparable to those found in the Cu (4.1(1) μ_B_) and Ag (3.99(2) μ_B_) oxyselenide analogues.

To confirm the nature of the metamagnetic behavior shown in the magnetometry, the NPD of the aged sample of Sr_2_MnO_2_Na_1.6_Se_2_ was collected at a vertical magnetic field of 5.5 T in 2023 (Figure 5c,d). As expected from the behavior of Sr_2_MnO_2_Cu_5.5_S_4_^10^ and the low-temperature magnetization isotherm (Figure 3b), the Bragg reflections characteristic of AFM ordering vanished, and extra intensity was observed on top of the nuclear Bragg peaks, suggesting that the compound is in the ferromagnetic regime. The magnetic contribution can be modeled with the magnetic space group *I*4/*mm*′*m*′ (139.537) in the BNS scheme. The mΓ3+ (a) mode gave a good fit to the data and produced a refined long-range-ordered moment of 3.3(1) μ_B_ (refined structural parameters in Table S3). This is close to the observed moment per Mn ion of 3.54 μ_B_ in the 2 K magnetization isotherm at 5.5 T and comparable to the long-range ordered moment found in the antiferromagnetic state using NPD. It should be noted that the magnetization isotherm was collected at 2 K and NPD at 5 K, although both are well below the Néel temperature. After this measurement, the field was ramped to zero, and another pattern was collected at 5 K (see Rietveld refinement in Figure S8). The zero-field NPD patterns collected before and after the application of the magnetic field looked similar. There was re-emergence of the magnetic reflections characteristic of the A-type antiferromagnetic state with similar intensity and no suggestion of field-induced preferred orientation (i.e., no changes in nuclear peak intensities; see Figure S9). A Rietveld refinement at 5 K in zero applied field was performed successfully by combining the patterns before and after the application of the magnetic field for better statistics. The refined parameters can be found in Table S2.

### Electrochemical Na Insertion

**Figure 6:**
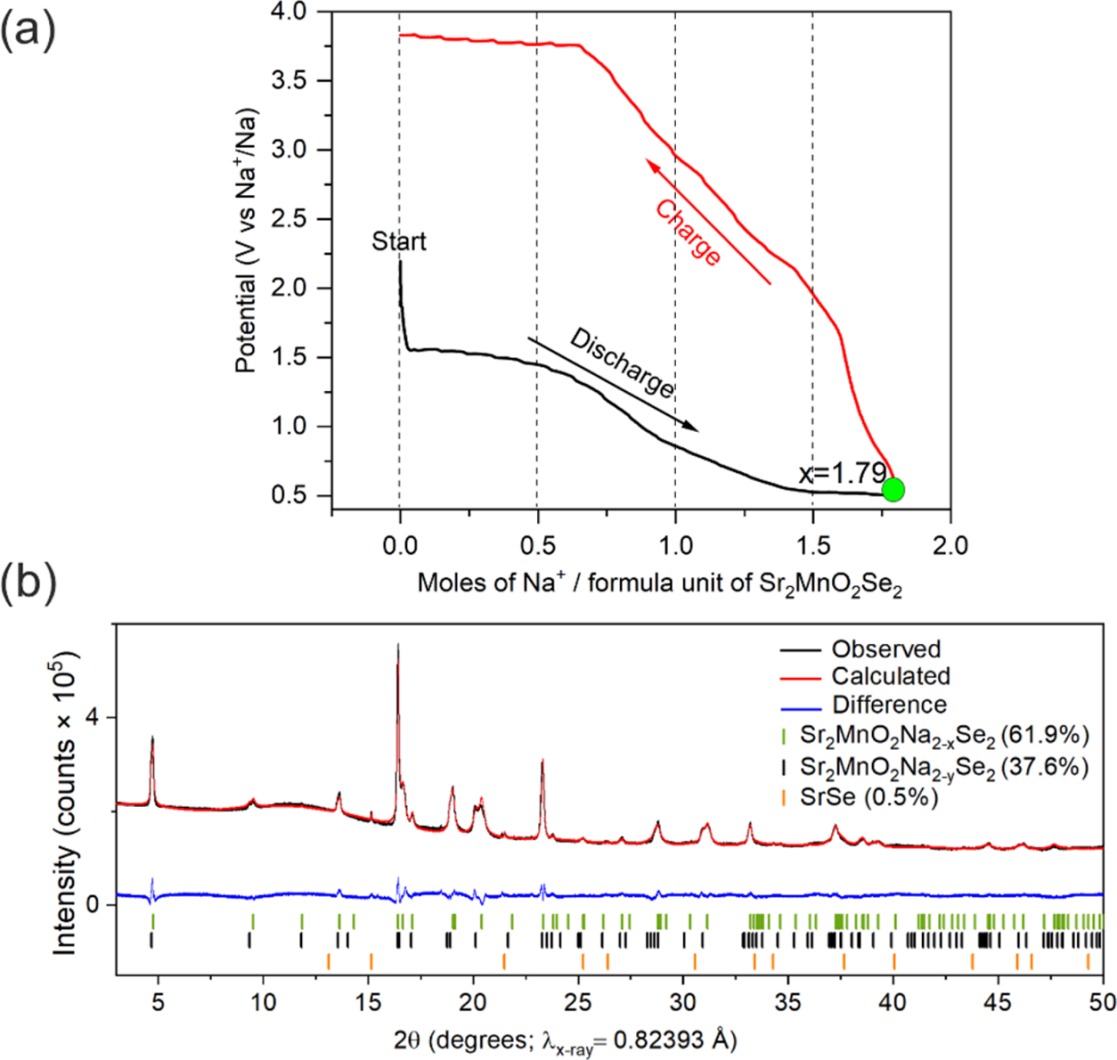
(a) Electrochemical Na intercalation in Sr_2_MnO_2_Se_2_. A 1.79 mol portion of Na can be intercalated per formula unit of Sr_2_MnO_2_Se_2_ according to the charge passed in the experiment. (b) Rietveld refinement against SPXRD data of the sample recovered after the end of discharge to 0.5 V. *R*_wp_ = 1.72% and χ^2^ = 6.54. The data can be fitted with two phases of Sr_2_MnO_2_Na_2–*x*_Se_2_ with slightly different lattice parameters and Na contents.

Sodium can be intercalated into the parent phase Sr_2_MnO_2_Se_2_ electrochemically. The voltage–composition profile of the Sr_2_MnO_2_Se_2_/Na cell is shown in Figure 6a. The sodiation of the layered oxyselenide is associated with a flat plateau at 1.5 V corresponding to the insertion of ∼0.5 Na per formula unit of oxyselenide, followed by a region with a sloping profile until the discharge cutoff voltage of 0.5 V. At the end of discharge at 0.5 V (vs Na^+^/Na), the sample gives a specific capacity of 114.2 mAh/g, which would correspond to 1.79 mol of Na being intercalated per formula unit of Sr_2_MnO_2_Se_2_, which is comparable to the Na content determined in a fresh sample obtained from the chemical intercalation. The presence of two different voltage steps indicates the occurrence of two different redox changes, presumably arising from the electron filling of Se–Se and Mn–Se hybridized states; however, determining the governing redox mechanism is beyond the scope of this work. The sample was isolated after discharge and measured using SPXRD. The diffraction data suggest a mixture of two phases, as there are shoulders next to many of the Bragg peaks. The data can be fitted with two Sr_2_MnO_2_Na_2–*x*_Se_2_ phases (space group: *I*4/*mmm*) with different lattice parameters (Figure 6b). Tentative refinement of the Na occupancies suggested a lower Na content of 0.74 (1) for the phase with the smaller cell volume (*a* = 4.077(0) Å, *c* = 19.837 (1) Å, *V* = 329.7(1) Å^3^) than for the phase with the larger cell volume (*a* = 4.086(0) Å, *c* = 20.223 (2) Å, *V* = 337.63 Å^3^; refined Na occupancy of 0.83(1)). We note that the facts that the electrochemically synthesized samples are multiphase, that Na makes a rather small contribution to the X-ray scattering, and that the data quality in Figure 6b is relatively low and insensitive to any amorphous material mean that we should be cautious about refining the composition of the two phases against these data. The cell volumes suggest that the electrochemically synthesized samples are actually slightly poorer in Na than even the aged chemically synthesized products. The comparison of electrochemically and chemically sodiated products is given in Table S4. The appearance of a two-phase mixture in the electrochemically synthesized sample implies that the electrochemical Na intercalation to reduce the Se–Se dimers is not entirely homogeneous. Anisotropic peak broadening using Stephens’s model^37^ was needed to fit the peaks.^31^

Preliminary data show that the charging process was accompanied by complete deintercalation of Na ions, albeit following a voltage path different from that of the discharge. Charging restores the Sr_2_MnO_2_Se_2_ phase (Figure S10), but there is a large hysteresis in the electrochemical cycling as observed for related systems.^20^ Future work will comprise the study of Na insertion at different current rates to reveal the redox mechanism with Na (de)insertion and in operando PXRD measurements to probe the phase and structural evolution during Na intercalation and deintercalation.^38^

## Conclusions

A new metastable transition metal oxychalcogenide phase Sr_2_MnO_2_Na_1.6_Se_2_ was synthesized by reductive sodium intercalation into the parent Sr_2_MnO_2_Se_2_ phase containing [Se_2_]^2–^ dimer units. The anion redox of these perselenide units is exploited to intercalate sodium and break Se–Se bonds to form [Na_2–*x*_Se_2_] antifluorite-type layers. Sodium naphthalenide has proven to be a chemoselective strong reductant to produce a pure product without parent phase degradation. The sodiated phase crystallizes in the space group *I*4/*mmm* and belongs to the Sr_2_MnO_2_Cu_1.5_S_2_ structural family. The structure is made of alternating [Sr_2_MnO_2_] cationic perovskite-type layers and [Na_2–*x*_Se_2_] anionic layers. The limiting composition obtained by this route has 16(1) % of the Na sites vacant in the chalcogenide layer from an NPD refinement, and this leads to a Mn formal oxidation state of +2.32. The sample is highly air-sensitive, and indeed, upon storing the sample in an argon glovebox atmosphere for two years, the Na content evolves slightly, with the Na vacancy content increasing to 20(1) % from the NPD refinement, the lattice parameters changing measurably, and the magnetic properties changing significantly. This behavior is comparable to the aerial deintercalation of about 5% of the Cu from the isostructural phase Sr_2_CoO_2_Cu_2_S_2_,^11^ although the Na-containing material is much more air-sensitive. The resulting increase in Mn oxidation takes it close to +2.5 with a Mn^2+^/Mn^3+^ ratio of 1:1. This leads to an increase in the ordered moment, presumably because of a decrease in the disorder in the magnetic interactions in the MnO_2_ plane. The Mn moments are long-range ordered at low temperatures with ferromagnetic MnO_2_ sheets coupled antiferromagnetically along the stacking axis. Application of magnetic fields >2 T overcomes the weak antiferromagnetic interactions, resulting in a metamagnetic transition to full ferromagnetic order. The critical field (*H*_M_) of the metamagnetic transition is inversely related to the interlayer spacing between adjacent MnO_2_ sheets in this class of compounds. Though Sr_2_MnO_2_Na_1.6_Se_2_ has a shorter interlayer spacing, it has a relatively low H_M_ compared with related compounds. Highly ionic bonding in the [Na_2–*x*_Se_2_] layer leads to a weaker bonding interaction between adjacent MnO_2_ sheets, thus lowering *H*_M_ and the Néel temperature for long-range magnetic ordering.

Sodium intercalation can also be performed reversibly using electrochemical means, though in this case, at the end of discharge, the product is a mixture of two sodiated phases with slightly different lattice parameters and estimated sodium content, and further analysis, including in operando structural measurements would be needed to determine the compositional ranges in the system Sr_2_MnO_2_Na_2–*x*_Se_2_ and how they correlate with electronic behavior.

This work highlights the value of multistep topochemical reactions to form new metastable phases with intriguing structural and magnetic properties.

## Supporting Information Available

Further diffractograms and tables of refined parameters and further magnetometry results (PDF)
Crystallographic information file for Sr_2_MnO_2_Na_1.6_Se_2_ (CIF)

## Supplementary Material

cm4c00801_si_001.pdf

cm4c00801_si_002.cif
