# Supplementary material for: Sr2MnO2Na1.6Se2: A Metamagnetic Layered Oxychalcogenide Synthesized by Reductive Na Intercalation to Break [Se2]2– Perselenide Dimer Units
Source: Chem Mater. 2024 May 30;36(11):5730–40. doi: 10.1021/acs.chemmater.4c00801 (PMC11171288; doi:10.1021/acs.chemmater.4c00801)
Supplement: Supplementary file 1 — cm4c00801_si_001.pdf [file cm4c00801_si_001.pdf]

$Sr_2MnO_2Na_{1.6}Se_2$: a metamagnetic layered oxychalcogenide synthesised by reductive Na intercalation to break $[Se_2]^{2-}$ perselenide dimer units

Souvik Giri[1], Sunita Dey[2], Emmanuelle Suard[3], and Simon J. Clarke[1]*

[1] Department of Chemistry, University of Oxford, Oxford OX1 3QR, UK.

[2] Department of Chemistry, University of Aberdeen, Meston Walk, Aberdeen, AB24 3UE, UK.

[3] Institut Laue-Langevin (ILL), BP 156, 71 Avenue des Martyrs, Grenoble 38042, France.

*email address: simon.clarke@chem.ox.ac.uk

Supporting Information

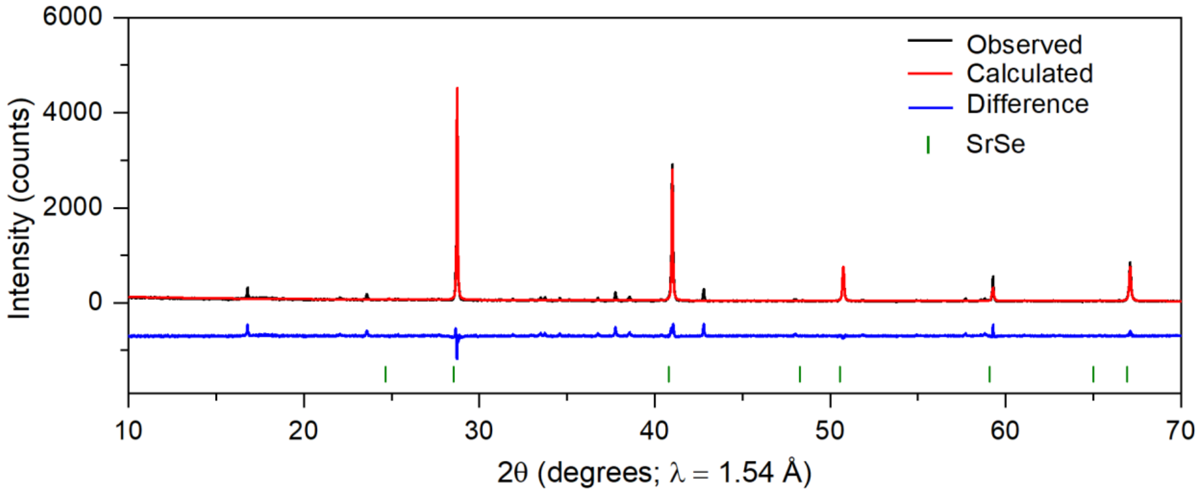

Fig S1. Powder X-ray diffraction pattern following an attempted high temperature synthesis of $Sr_2MnO_2Na_{1.6}Se_2$ using the reaction mixture: 2SrO + Mn + Na_2Se + Se. The reactants were ground, made into a pellet and heated in an alumina crucible inside a sealed, evacuated silica ampule. The sample was heated to 850 °C at 5 °C min^{-1}, held at this temperature for 16 hrs and cooled at the natural rate of the furnace. The pattern was dominated by SrSe, showing that the target phase was not formed and that low-temperature synthesis is required. Other Bragg peaks were not indexed and were presumed to arise from several Na, Mn and O-containing phases.

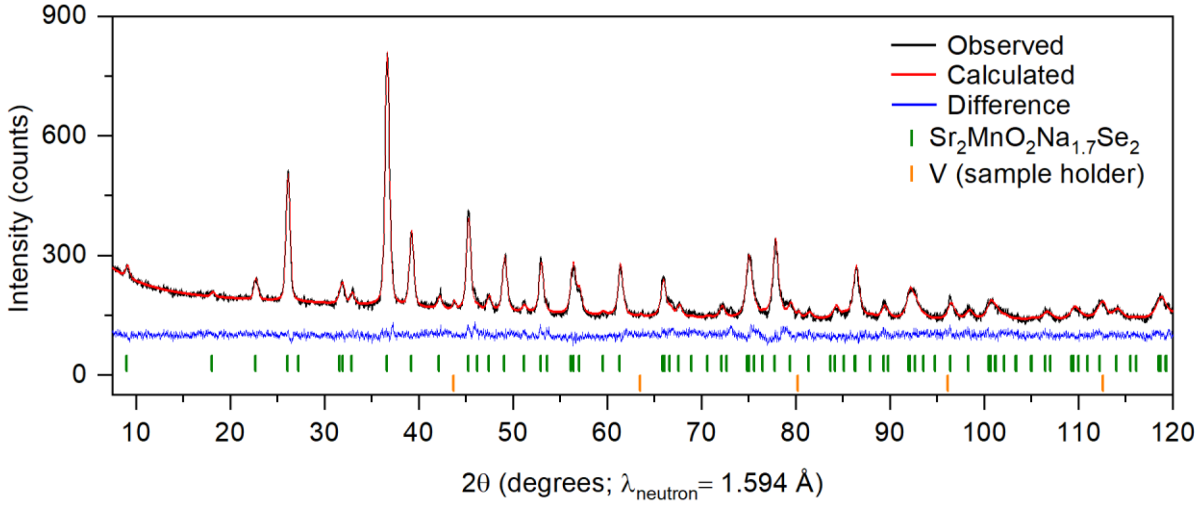

Fig S2: Rietveld refinement of the fresh $Sr_2MnO_2Na_{1.7}Se_2$ sample against NPD data (collected in 2021) at room temperature. R_{wp} = 3.5 % and χ^2 = 1.16. In this case, the composition is represented as $Sr_2MnO_2Na_{1.7}Se_2$ (different from the aged version of the same sample ($Sr_2MnO_2Na_{1.6}Se_2$) described in the main text), which reflects the Na content refined from this data.

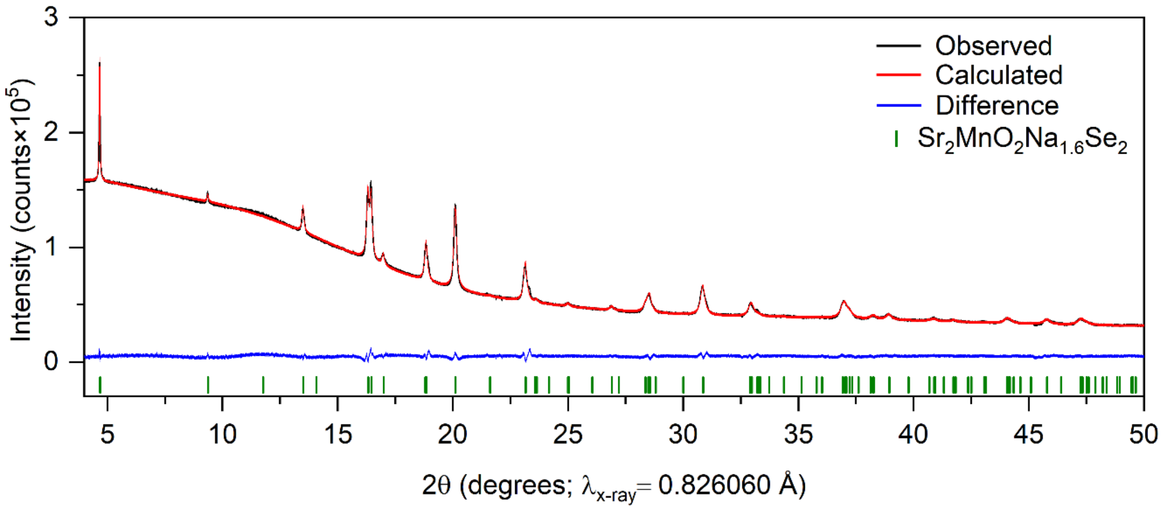

Fig S3: Rietveld refinement of the $Sr_2MnO_2Na_{1.6}Se_2$ intercalant against SPXRD data at 100 K collected in May 2023. R_{wp} = 1.07 % and χ^2 = 2.81. The pattern can be modelled with the room temperature model of $Sr_2MnO_2Na_{1.6}Se_2$ and accounting for lattice parameter contraction. No super-structure peaks were present, which suggests the absence of Na vacancy ordering.

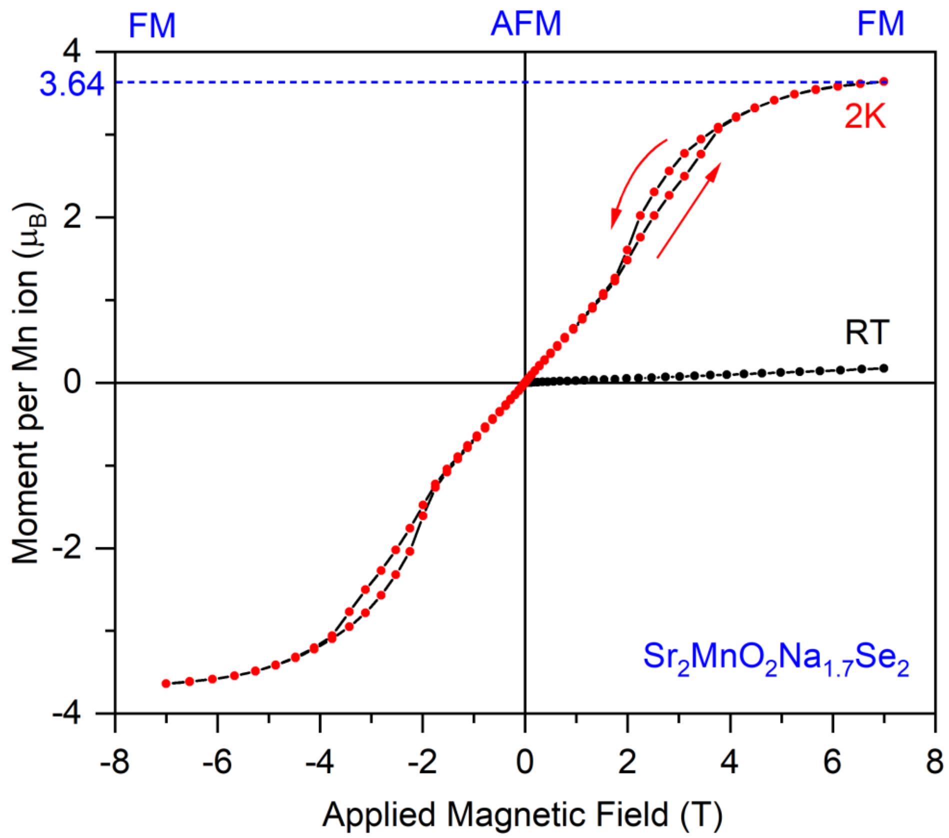

Fig S4: Magnetisation(M) vs magnetic field (H) isotherm at RT and 2 K for $Sr_2MnO_2Na_{1.7}Se_2$.

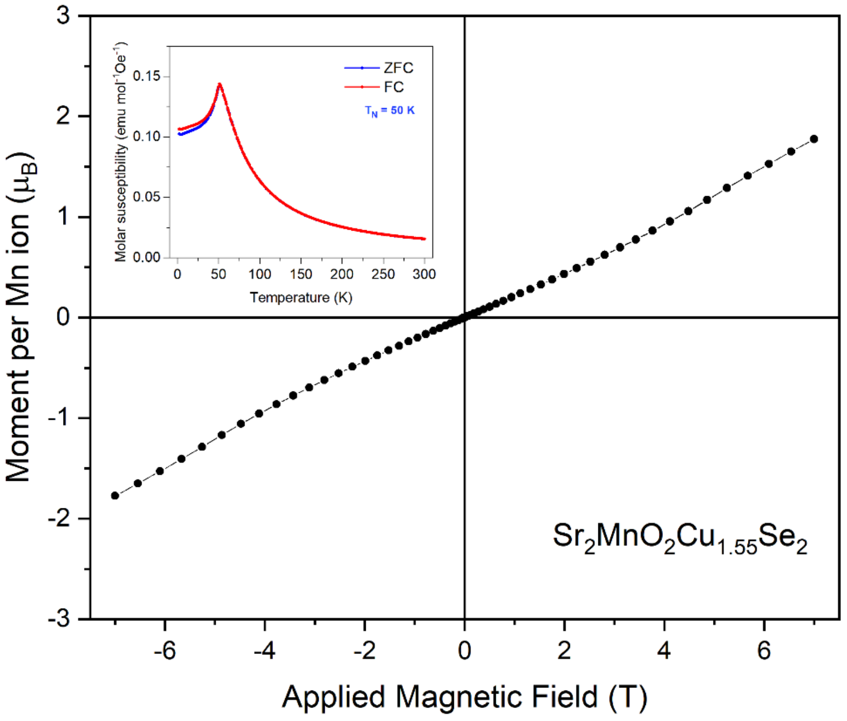

Fig S5: Magnetisation(M) vs magnetic field (H) isotherm at 2 K for $Sr_2MnO_2Cu_{1.55}Se_2$ with a slight upturn at high fields. The inset shows M vs T for the same in ZFC and FC conditions.

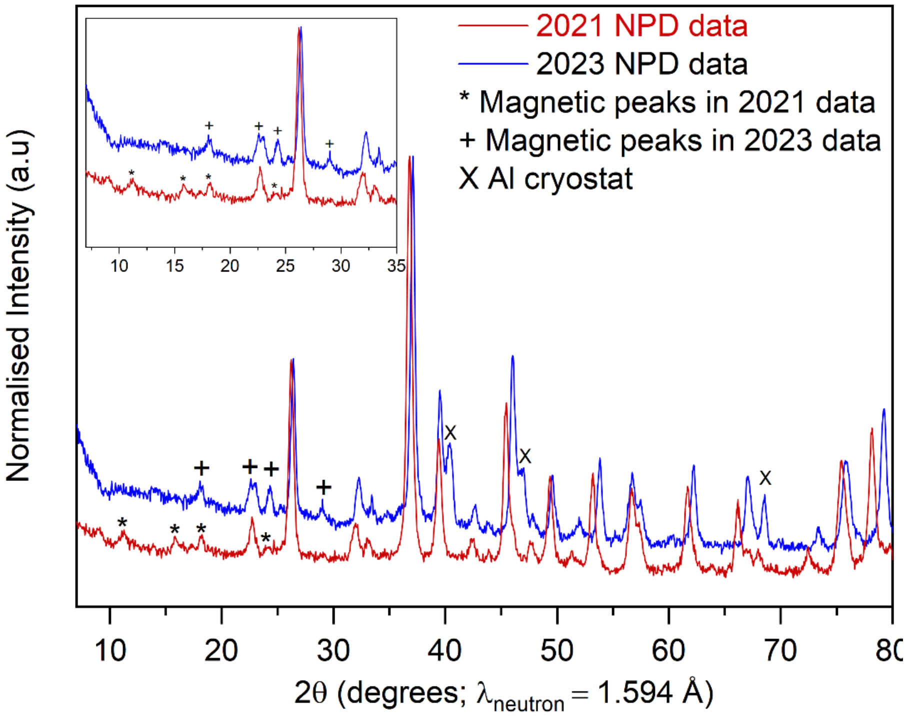

Fig S6: Comparison of NPD data collected on the same sample of $Sr_2MnO_2Na_{2-x}Se_2$ at 2 K in 2021 and at 5 K (and zero applied magnetic field) in 2023. The shift of nuclear peaks to a higher angle is in line with a slight change in composition and lattice parameters over two years. The inset shows the different nature of magnetic scattering in two data sets. The Al peaks in the 2023 data and the higher background are a consequence of the cryomagnet sample environment used for that experiment.

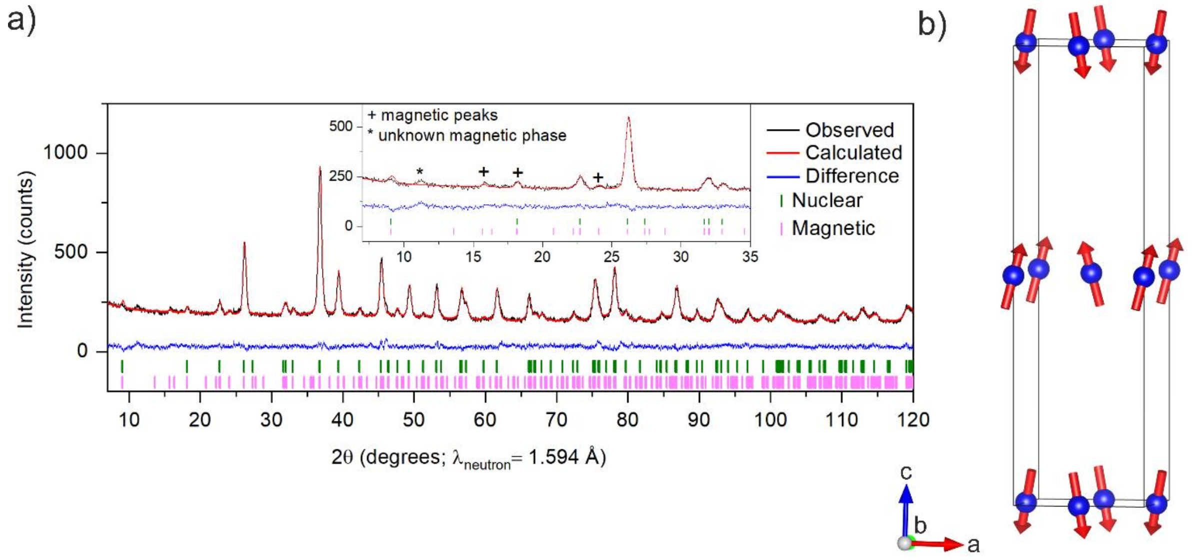

Fig S7: a) Rietveld refinement against PND data at 2 K collected in 2021. R_{wp} = 3.65 % and χ^2 = 1.25. The inset shows the fit of the magnetic model at low angle. b) The refined magnetic model from Rietveld refinement. Only Mn ions are shown for clarity. The magnetic structure can be modelled as ferromagnetic MnO_2 planes with Mn moments tilted away from the crystallographic *c* axis, coupled antiferromagnetically.

Table S1: $Sr_2MnO_2Na_{1.7}Se_2$ structural parameters at 2 K from NPD data collected in 2021

Compound	$Sr_2MnO_2Na_{1.7}Se_2$
Radiation and wavelength	Neutron, 1.594 Å
Date of measurement	October, 2021
Temperature and magnetic Field	5 K, 0 T
Spacegroup	*I4/mmm* (No: 139)
a (Å)	4.1349 (1)
c (Å)	20.212 (1)
V (Å^3)	345.592 (3)
z(Sr)*	0.08110 (1)
z(Se)*	0.1516(1)
Na occupancy	0.83 (1)
B_{iso}(Sr) (Å^2)	0.45 (2)
B_{iso}(Mn) (Å^2)	0.45 (2)
B_{iso}(O) (Å^2)	0.45 (2)
B_{iso}(Na) = B_{iso}(Se)×2 (Å^2)	0.92 (5)
B_{iso}(Se) (Å^2)	0.45 (2)
Magnetic Spacegroup	$P4_2'/ncm'$ (138.523) in the BNS scheme
Refined magnetic moment (μ_B)	2.04
R_{wp} (%)	3.65

*Sr, 4*e*(0,0,*z*); Mn, 2*a*(0,0,0); O, 4*c*(½,0,0); Na, 4*d*(½,0,¼); Se, 4*e*(0,0,*z*)

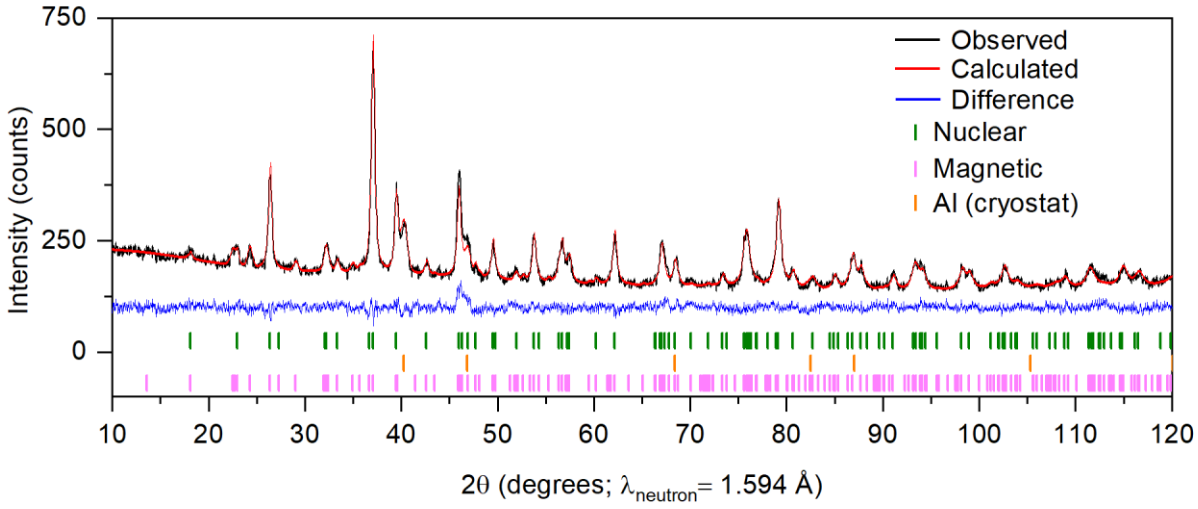

Fig S8: Rietveld refinement against PND data at 5 K after turning off the magnetic field R_{wp} = 3.51 % and χ^2 = 1.31. These data were collected on the aged sample $Sr_2MnO_2Na_{1.6}Se_2$ in 2023.

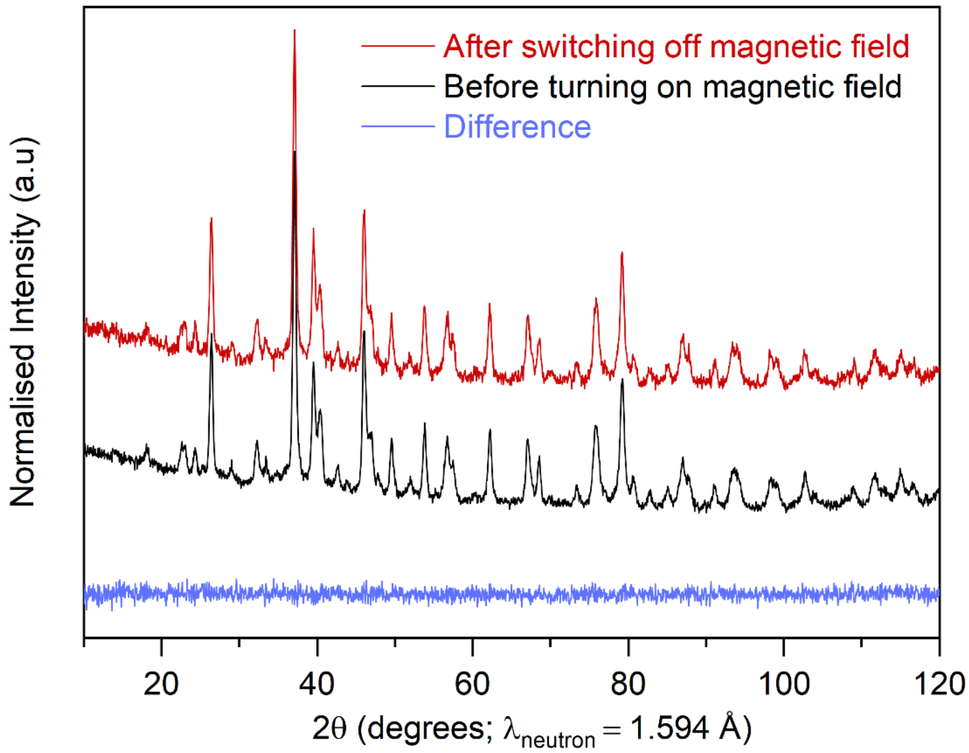

Fig S9: Comparison of the zero-field NPD patterns collected before and after the application of the magnetic field. The flat difference between the normalised data sets suggests no field-induced preferred orientation of particles. The lower signal-to-noise in the NPD pattern measured after switching off the magnetic field is due to the shorter data collection time compared to the NPD pattern measured before applying the magnetic field. These data were collected on the aged sample $Sr_2MnO_2Na_{1.6}Se_2$ in 2023 in the cryomagnet sample environment.

Table S2: $Sr_2MnO_2Na_{1.6}Se_2$ structural parameters at 5 K from combined Rietveld refinement of NPD data at 5 K before and after application of magnetic field

Compound	$Sr_2MnO_2Na_{1.6}Se_2$
Radiation and wavelength	Neutron, 1.594 Å
Date of measurement	November, 2023
Temperature and magnetic Field	5 K, 0 T
Magnetic Spacegroup	P_I4/mnc (128.410) in the BNS scheme
a (Å)	4.0818 (1)
c (Å)	20.247 (1)
V (Å^3)	337.35 (4)
z(Sr)*	0.08247 (1)
z(Se)*	0.1491(1)
Na occupancy	0.71 (1)
B_{iso}(Sr) (Å^2)	0.10 (3)
B_{iso}(Mn) (Å^2)	0.10 (3)
B_{iso}(O) (Å^2)	0.10 (3)
B_{iso}(Na) = B_{iso}(Se)×2 (Å^2)	0.21 (6)
B_{iso}(Se) (Å^2)	0.10 (3)
Refined magnetic moment (μ_B)	3.79 (9)
R_{wp} (%)	3.47

*Sr, 4*e*(0,0,*z*); Mn, 2*a*(0,0,0); O, 4*c*(½,0,0); Na, 4*d*(½,0,¼); Se, 4*e*(0,0,*z*)

Table S3: $Sr_2MnO_2Na_{1.6}Se_2$ structural parameters from Rietveld refinement of NPD data at 5 K at 5.5 T magnetic field

Compound	$Sr_2MnO_2Na_{1.6}Se_2$
Radiation and wavelength	Neutron, 1.594 Å
Date of measurement	November, 2023
Temperature and magnetic Field	5 K, 5.5 T
Magnetic Spacegroup	*I4/mm'm'* (139.537) in the BNS scheme
a (Å)	4.0808 (1)
c (Å)	20.239 (2)
V (Å^3)	337.059 (4)
z(Sr)*	0.0822(1)
z(Se)*	0.1492 (1)
Na occupancy	0.76 (2)
B_{iso}(Sr) (Å^2)	0.41 (4)
B_{iso}(Mn) (Å^2)	0.41 (4)
B_{iso}(O) (Å^2)	0.41 (4)
B_{iso}(Na) = B_{iso}(Se)×2 (Å^2)	0.83 (8)
B_{iso}(Se) (Å^2)	0.41 (4)
Refined magnetic moment (μ_B)	3.3 (1)
R_{wp} (%)	3.07

*Sr, 4*e*(0,0,*z*); Mn, 2*a*(0,0,0); O, 4*c*(½,0,0); Na, 4*d*(½,0,¼); Se, 4*e*(0,0,*z*)

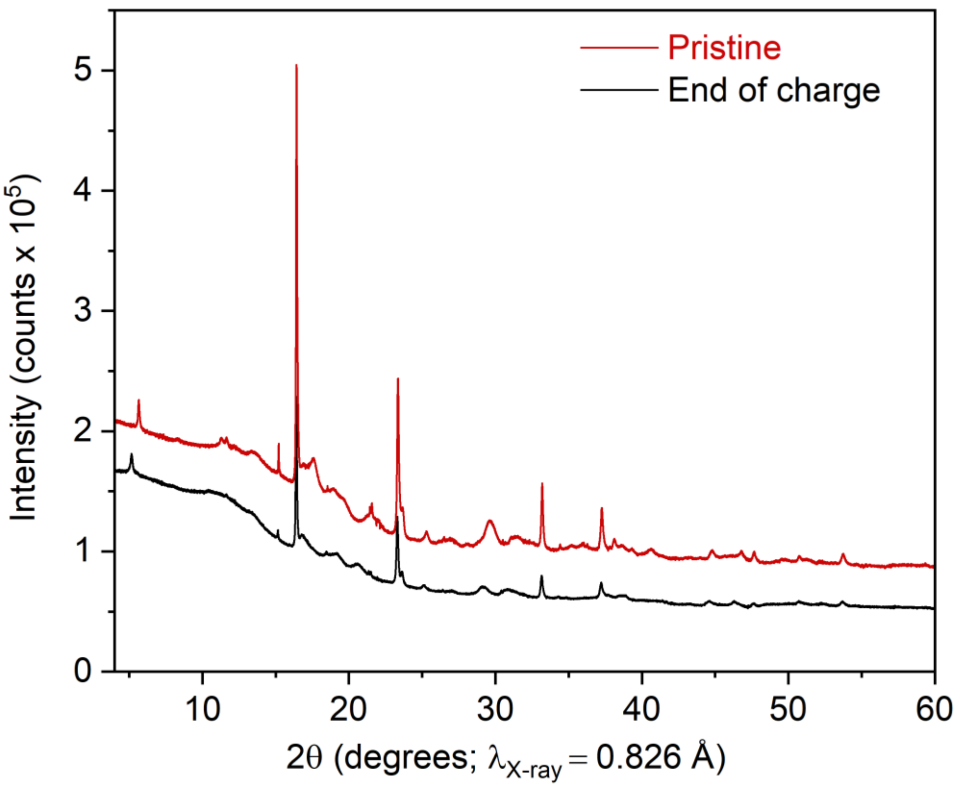

Fig S10: Comparison of the SPXRD patterns collected for the pristine sample of $Sr_2MnO_2Se_2$ and the sample recovered after the end of charging to 3.8 V. The similarity of the two powder patterns suggests that at the end of charge, the $Sr_2MnO_2Se_2$ phase is restored but with lower crystallinity.

Table S4: Comparison of structural parameters from electrochemical and chemical Na intercalation

	Electrochemical		Chemical
	Phase 1	Phase 2	
Radiation		Synchrotron X-ray	
Space group		*I*4/*mmm* (139)	
a (Å)	4.0770 (1)	4.0862(1)	4.10529(4)
c (Å)	19.837 (1)	20.223 (2)	20.3419(4)
c/a	4.8656 (3)	4.9491 (5)	4.9550 (1)
V (Å^3)	329.73 (4)	337.67 (5)	342.83 (1)
Na occupancy	0.737 (9)	0.83 (1)	0.829 (4)
Weight fraction (%)	61.9	37.6	100
